# Origin and diversification of leucine-rich repeat receptor-like protein kinase (*LRR-RLK*) genes in plants

**DOI:** 10.1186/s12862-017-0891-5

**Published:** 2017-02-07

**Authors:** Ping-Li Liu, Liang Du, Yuan Huang, Shu-Min Gao, Meng Yu

**Affiliations:** 1 College of Biological Sciences and Biotechnology, Beijing Forestry University, Beijing, 100083 China; 2 College of Life Sciences, Peking University, Beijing, 100871 China

**Keywords:** *LRR-RLK* genes, Functional divergence, Gene structure, Motif, Positive selection

## Abstract

**Background:**

Leucine-rich repeat receptor-like protein kinases (LRR-RLKs) are the largest group of receptor-like kinases in plants and play crucial roles in development and stress responses. The evolutionary relationships among *LRR-RLK* genes have been investigated in flowering plants; however, no comprehensive studies have been performed for these genes in more ancestral groups. The subfamily classification of *LRR-RLK* genes in plants, the evolutionary history and driving force for the evolution of each *LRR-RLK* subfamily remain to be understood.

**Results:**

We identified 119 *LRR-RLK* genes in the *Physcomitrella patens* moss genome, 67 *LRR-RLK* genes in the *Selaginella moellendorffii* lycophyte genome, and no *LRR-RLK* genes in five green algae genomes. Furthermore, these *LRR-RLK* sequences, along with previously reported *LRR-RLK* sequences from *Arabidopsis thaliana* and *Oryza sativa*, were subjected to evolutionary analyses. Phylogenetic analyses revealed that plant *LRR-RLK*s belong to 19 subfamilies, eighteen of which were established in early land plants, and one of which evolved in flowering plants. More importantly, we found that the basic structures of *LRR-RLK* genes for most subfamilies are established in early land plants and conserved within subfamilies and across different plant lineages, but divergent among subfamilies. In addition, most members of the same subfamily had common protein motif compositions, whereas members of different subfamilies showed variations in protein motif compositions. The unique gene structure and protein motif compositions of each subfamily differentiate the subfamily classifications and, more importantly, provide evidence for functional divergence among *LRR-RLK* subfamilies. Maximum likelihood analyses showed that some sites within four subfamilies were under positive selection.

**Conclusions:**

Much of the diversity of plant *LRR-RLK* genes was established in early land plants. Positive selection contributed to the evolution of a few *LRR-RLK* subfamilies.

**Electronic supplementary material:**

The online version of this article (doi:10.1186/s12862-017-0891-5) contains supplementary material, which is available to authorized users.

## Background

All living organisms sense and conduct signals through cell surface receptors. In plants, many such cellular signaling transductions are mediated by receptor-like kinases (RLKs). The largest group of plant RLKs is the leucine-rich repeat RLK family (LRR-RLK) [1]. LRR-RLKs contain three functional domains: an extracellular domain (ECD) that perceives signals, a transmembrane domain that anchors the protein within the membrane, and an intracellular kinase domain (KD) that transduces the signal downstream via autophosphorylation, followed by subsequent phosphorylation of specific substrates [2]. The LRR-RLK ECD contains varying numbers of LRR repeats, and LRR diversity enables LRR-RLKs to sense a variety of ligands, including small molecules, peptides, and entire proteins [3]. On the other hand, the LRR-RLK KD is common in protein kinases, and contains 12 conserved subdomains that fold into a similar three-dimensional catalytic core with a two-lobed structure [4, 5]. Previous investigations demonstrated that all conserved residues in these subdomains play essential roles in enzyme function [4, 5].

LRR-RLKs function in a wide array of plant processes. Some LRR-RLKs are involved in the control of plant growth and development; for example, CLV1 is involved in controlling meristem development [6, 7], RUL1 is involved in secondary growth [8], SERK1 is involved in microsporogenesis and embryogenesis [9], and BRI1 is involved in brassinosteroid signaling [10]. Some LRR-RLKs respond to abiotic and biotic stresses, such as FLS2- and EFR-mediated plant resistance against bacterial pathogens [11, 12], and NIK activity in antiviral defense [13, 14]. Some *LRR-RLK* genes have dual roles in development and defense due to cross-talk between these two pathways or recognition of multiple ligands by the same receptor [15]. For example, BAK1 is involved in developmental regulation through interaction with the plant brassinosteroid receptor BRI1, and it is involved in innate immunity against pathogens through interaction with FLS2, which recognizes the flg22 peptide from bacterial flagellin. *LRR-RLK* genes have been extensively studied and the results show that they have crucial roles in plant development and stress responses. However, there are numerous *LRR-RLK* genes, and the functions of the vast majority of them are largely unknown.

Evolutionary studies of genes can provide insights into possible gene functions and mechanisms of gene duplication and functional divergence. With regard to the evolution of *LRR-RLK* genes, investigations have been only performed in flowering plants [1, 16–23]. Several questions about the evolutionary history of *LRR-RLK* genes remain to be answered. First, how many *LRR-RLK* gene subfamilies can be classified in plants, and when did each subfamily originate? Based on the phylogenetic relationships of kinase domains and the arrangement of LRR motifs, *LRR-RLK* genes were classified into 15 groups in *Arabidopsis thaliana* [1], 5 groups in *Oryza sativa* [17] and 14 groups in *Populus trichocarpa* [18]. The phylogenetic analysis for each classification was based on *LRR-RLK* genes from the same species; therefore, these studies provide a useful but limited phylogenetic framework for the classification of these genes in plants. Nevertheless, previous studies did not elucidate the origin of each subfamily due to the lack of phylogenetic analysis of *LRR-RLK* genes from diverse plants, including algae, bryophytes, and different lineages of vascular plants.

Second, it is not known how *LRR-RLK* intron/exon structures and protein sequences evolved accompanying the plant evolution. Protein sequences and motifs are directly related to protein function. Introns have important roles in cellular and developmental processes via alternate splicing or gene expression regulation [24]. The presence of multiple introns is essential for the expression of the *ERECTA LRR-RLK* gene in *A. thaliana* [25]. Analysis of the intron/exon structures and protein sequences of different *LRR-RLK* subfamilies is important to understand the evolution of gene function among the subfamilies [26]. Earlier studies provided important clues on the evolution of the intron/exon structures and protein motifs of the *LRR-RLK* genes from flowering plants [17, 18]. For example, *LRR-RLK* genes within the same subfamily usually have similar intron/exon structures and protein motifs, while members of different subfamilies exhibit different genomic structures and protein motifs [16–22]. However, it is unknown whether these patterns would be consistent if more basal plants were analyzed. Furthermore, in terms of gene structures, previous studies did not reveal when the common structure of each subfamily was established and how these structures evolved along different major plant lineages.

Finally, what was the evolutionary force driving the evolution of each *LRR-RLK* subfamily? Genes accumulate mutations during evolution, and this may be due to a relaxation of purifying selection or the action of positive selection [27, 28]. Positive selection has been detected in many duplicated genes [29–34]. Previous studies demonstrated that positive selection contributed to the evolution of some *LRR-RLK* subfamilies defined in *A. thaliana* and *O. sativa* [17, 35–38]. A recent study demonstrated that selection constraint appeared to be globally relaxed at lineage-specific expanded *LRR-RLK* genes, of which 50% contained codons under positive selection [23]. In this study, we try to investigate how many *LRR-RLK* subfamilies defined in the present phylogenetic analysis were controlled by positive selection, and evaluated the relative importance of relaxation of purifying selection and positive selection in the evolution of *LRR-RLK* subfamilies.

The complete genome sequences from different major plant lineages now available allow us to examine the evolutionary history of *LRR-RLK* genes in plants. Previous studies have identified *LRR-RLK* genes mainly from flowering plants [1, 17–23]. In this study, we identified *LRR-RLK* sequences in the complete genomes of representative species of other major plant lineages, including four completely sequenced green alga species (*Chlamydomonas reinhardtii*, *Micromonas pusilla* CCMP1545 and *Micromonas* sp. RCC299, *Ostreococcus lucimarinus*, and *Volvox carteri*), one moss species (*Physcomitrella patens*), and one lycophyte species (*Selaginella moellendorffii*). Next, these sequences and previously identified sequences in two flowering plants (*A. thaliana* and *O. sativa*) [1, 17] were subjected to phylogenetic analysis, gene structure and motif determination, and evolutionary pressure analysis. The objectives of this study are : (1) to classify *LRR-RLK* subfamilies in divergent plant species and determine the origin of each subfamily, (2) to determine the evolutionary history of gene structures and the evolutionary patterns of the protein sequences of each subfamily, and (3) to evaluate potential selection pressure that promoted the evolution of each *LRR-RLK* subfamily.

## Methods

### Identification of *LRR-RLK* gene sequences

The *Arabidopsis thaliana LRR-RLK* sequences reported by Shiu et al. [1] were retrieved from ‘The *Arabidopsis* Information Resource’ (TAIR, http://www.arabidopsis.org/) [39]. The *Oryza sativa LRR-RLK* sequences were obtained from a previous study [17]. The kinase domain sequences of representative proteins from each *LRR-RLK* subfamily of *A. thaliana* were used as queries to conduct Blastp searches (E-value cutoff < 1 × 10^−10^) against the protein databases of six species available on Phytozome v11.0 [40]. The six species are representative of major plant lineages other than flowering plants, including four fully sequenced green alga species (*Chlamydomonas reinhardtii*, *Micromonas pusilla* CCMP1545 and *Micromonas* sp.RCC299, *Ostreococcus lucimarinus*, and *Volvox carteri*), one moss species (*Physcomitrella patens*), one lycophyte species (*Selaginella moellendorffii*). The resulting hits were downloaded from Phytozome v11.0. Identical and defective sequences were identified and eliminated by manual inspection in BioEdit [41]. Potential kinase sequences were analyzed with Pfam (http://pfam.xfam.org/) [42] and SMART (http://smart.embl-heidelberg.de/) [43] to confirm the presence of at least one LRR domain (PF00560) and one KD domain (PF00069), after which they were analyzed with TMHMM v. 2.0 (http://www.cbs.dtu.dk/services/TMHMM/) [44] to confirm the presence of transmembrane domains (TMs). Sequences were considered to be *LRR-RLK*s if they contained LRRs in the ECD, TMs, and a KD [45]. No *LRR-RLK* genes were identified in four fully sequenced green alga species. Therefore, only *LRR-RLK* genes identified in the genomes of *P. patens* and *S. moellendorffii* were used for further analysis. Our preliminary studies found that the *LRR-RLK* genes identified in the *P. patens* genome version 3.3 were well annotated. However, the annotations of some *LRR-RLK* genes in the *S. moellendorffii* genome version 1.0 had some problems according to the analysis of sequence homology and gene structure. To prevent the inclusion of falsely annotated data that could bias our analyses, we manually re-annotated the problematic *LRR-RLK* genes from *S. moellendorffii* using available expression data and sequence similarities with the homologous genes.

After *LRR-RLK* sequences were obtained, we compared the proportions of *LRR-RLK* genes among all protein-coding genes for different genomes. The numbers of *LRR-RLK* genes contained in the genomes of angiosperm species were obtained from published papers [1, 17–22]. The number of protein-coding genes in each genome was obtained from Phytozome v11.0.

### *LRR-RLK* gene alignments and phylogenetic analysis

*LRR-RLK* sequences obtained in the present study and previously reported in *A. thaliana* and *O. sativa* [1, 17] were used in the phylogenetic analysis. Raf kinase (At1g18160) and Aurora kinase (At2g25880) were defined as outgroups, similarly as in a previous study [46]. Multiple sequence alignments were performed with Muscle [47], after which they were manually adjusted in BioEdit [41]. Sequences outside of the kinase domain were deleted because their alignments were ambiguous. The amino acid sequences of the KDs were subjected to phylogenetic analysis. Phylogenetic trees were constructed using the maximum likelihood (ML) method implemented in RAxML 7.2.6 [48]. The best-fit evolutionary model (JTT amino acid substitution model) was selected using the Akaike information criterion in ProtTest version 3 [49]. The starting tree was obtained with BioNJ, and parameter values were estimated from the data. Branch support was estimated from 1000 bootstrap replicates.

### Analysis of gene structure and conserved motifs

To study intron evolution, the intron/exon structures for each gene were mapped to their corresponding genes. The structures of most *LRR-RLK* genes were retrieved from the Phytozome v11.0. The intron/exon structures of some re-annotated sequences were determined by comparing their CDS with their corresponding genomic DNA sequences, after which these structures were displayed using the Gene Structure Display Server (GSDS) (http://gsds.cbi.pku.edu.cn/) [50]. The gene structures were positioned in front of the phylogenetic tree. For each subfamily, the proportion of genes containing a given intron and the proportion of genes with a given gene structure were calculated. To elucidate the protein sequence evolution, the LRR domain and conserved KD motifs were identified with the Multiple Expectation Maximization for Motif Elicitation (MEME) program v.4.10.2. (http://alternate.meme-suite.org/) [51]. Due to a limitations on the maximum number of characters, the kinase domain data set was separated into three data sets from the N-terminus to C-terminus to perform MEME analysis. The MEME parameters for the KD data sets were as follows: the maximum number of motifs for the first and second data sets, 5; the maximum number of motifs for the third data set 10; minimum motif width, 10; and maximum motif width, 30; and all other parameters were defaulted. The MEME parameters for the LRR domain data were set as follows: the maximum number of motifs, 20; motif width, 24 (because the length of the plant LRR is 24 amino acids).

### Test for evolutionary selection pressure

The nonsynonymous/synonymous rate ratio (ω = d_N_/d_S_) is an effective measure to detect selection on protein-coding genes: ω = 1, neutral evolution; ω < 1, purifying selection; and ω > 1, positive selection. To evaluate the selective pressures acting on the *LRR-RLK* genes in each subfamily, we estimated the ω value of each subfamily using a maximum likelihood method. Previous studies demonstrated that the positive selection pressure acting on orthologs and paralogs differs in extent [23, 52]. Therefore, the ω values of the orthologs and paralogs of each subfamily were estimated separately as reported in Fischer et al. [23]. First, we identified ultraparalog (UP; related only by duplication) clusters and superortholog (SO; related only by speciation) clusters as reported in Fischer et al. [23] using a tree reconciliation approach [53]. Next, we estimated the ω values of the UP and SO clusters of each subfamily using the codeml program in the PAML 4.8 package [54]. Only clusters with a minimum of five sequences were assessed with the codeml site-model. The codon alignments used as input for codeml were created with DAMBE [55]. The phylogenetic trees for codeml were reconstructed by PhyML 3.0 [56] under the GTR substitution model. Six site models (model = 0; NSsites = 0, 1, 2, 3, 7, 8) were performed for each cluster. The M0 model assumes the same ω for all branches and all sites, whereas the M3 model uses a general discrete distribution with three site classes. We conducted likelihood ratio tests (LRTs) of the log likelihood (InL) of the M0 and M3 models to test for variable selective pressure among sites. The nearly neutral model (M1) assumes sites with ω ≤ 1, while the positive selection model (M2) is an extensive of M1 and assumes a third class of positive-selected sites (ω > 1). The beta model (M7) assumes a beta distribution for the ratio over sites, whereas the beta&ω model (M8) adds an extra class of sites with ω > 1 to the M7 model. Two pairs of nested models (M1a/M2a and M7/M8) were compared using LRTs to test for evidence of sites evolving by positive selection.

## Results

### Phylogenetic analysis of *LRR-RLK* genes

No *LRR-RLK* genes were identified in five completely sequenced genomes of green alga species; however, we identified 119 *LRR-RLK* genes in the *Physcomitrella patens* moss genome and 67 *LRR-RLK* genes in the *Selaginella moellendorffii* lycophyte genome (Additional file 1: Table S1). We calculated the proportions of *LRR-RLK* genes among all protein-coding gene in these two species and eight angiosperm species. The proportions of *LRR-RLK* genes in moss and lycophytes are 0.36 and 0.30%, respectively, while the proportions of *LRR-RLK* genes in the eight angiosperm species are 0.67–1.39% (Table 1).

**Table 1:** Percentage of *LRR-RLK* genes among all protein-coding genes

Species	Number of *LRR-RLK* genes ^[References]^	Number of protein-coding genes	Percentage (%)
Physcomitrella patens	119	32,926	0.36
Selaginella moellendorffii	67	22,273	0.30
*Oryza sativa*	309 [17]	22,273	1.39
*Arabidopsis thaliana*	213 [1]	27,416	0.78
*Brassica rapa*	303 [20]	40,492	0.75
C*itrus clementina*	300 [22]	24,533	1.22
*Citrus sinensis*	297 [22]	25,376	1.17
*Glycine max*	467 [21]	56,044	0.83
*Populus trichocarpa*	379 [18]	41,335	0.92
*Solanum lycopersicum*	234 [19]	34,727	0.67

**Fig. 1:**
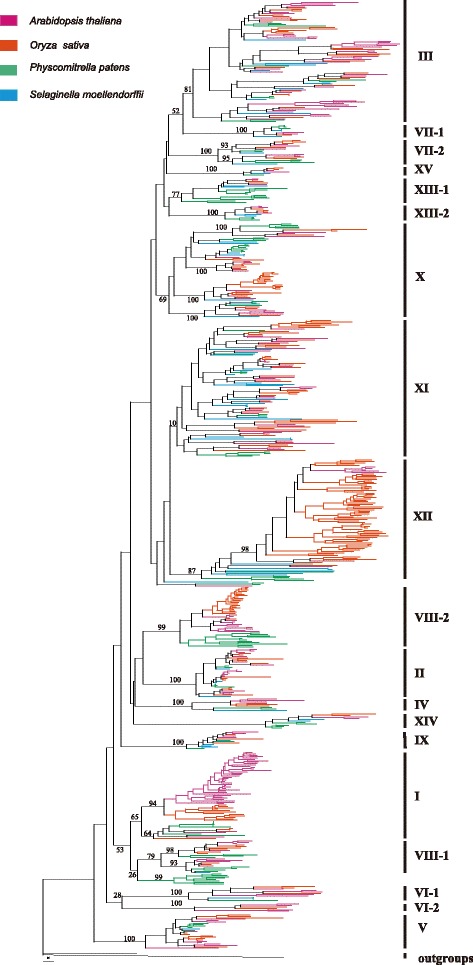
Phylogenetic tree of *LRR-RLK* genes. The phylogenetic tree was constructed by the maximum likelihood method and based on kinase domain amino acid sequences with sequences from *Physcomitrella patens*, *Selaginella moellendorffii*, *Arabidopsis thaliana*, and *Oryza sativa*. Bootstrap values of major clades are shown above branches. The subfamily names are shown on the right. The full phylogeny is shown in Additional file 2: Figure S1

We combined *LRR-RLK* sequences identified in the present study with previously reported *LRR-RLK* sequences from *A. thaliana* and *O. sativa* to generate a primary data set. The alignment of the *LRR* region is ambiguous, so only conserved kinase domain regions were used for the phylogenetic analysis (Additional file 5: Data S1﻿). Phylogenetic trees were constructed by maximum likelihood (ML). As shown in the ML tree (Fig. 1 and Additional file 2: Figure S1), the *LRR-RLK* genes clearly fell into distinct clades, indicating that these natural groups can be assigned to different subfamilies. These subfamilies are mostly consistent with the groups proposed by previous phylogenetic and structural analyses of *A.thaliana LRR-RLK* genes [1]. Therefore, we adopted the *A.thaliana LRR-RLK* group nomenclature proposed by Shiu and Bleecker [1] to label these subfamilies, with a few modifications: for example, subfamilies VI, VII, and XIII were subdivided into subfamilies VI-1 and VI-2; VII-1, and VII-2, and XIII-1 and XIII-2, respectively. In total, *LRR-RLK* genes were divided into 19 different subfamilies (Fig. 1). All subfamilies except XI were supported as clades with moderate to high bootstrap support (65–100%). For group XI, the topology varied between trees: either the group XI appears to be a monophyletic clade with very low branch support (<50%, Fig. 1) or paraphyletic (tree not shown). As we could not confirm that group XI was monophyletic, it was omitted from further analysis. Of the 19 LRR-RLK subfamilies (Fig. 1), subfamily VI-2 did not include sequences from *P. patens* and *S. moellendorffii*; subfamilies I, and VIII-2 did not include sequences from *S. moellendorffii*; and all other subfamilies included *LRR-RLK* sequences from all four species. In addition, a clade composed of eight *P. patens LRR-RLK* genes is a sister clade to subfamily VIII-1. However, we did not include these *P. patens LRR-RLK* genes into the subfamily VIII-1 as this relationship was not strongly supported. Nevertheless, these *P. patens* genes are phylogenetically closest to subfamily VIII-1. This clade probably represents a group that evolved in *P. patens* or, alternatively, was present in the common ancestors of land plants and lost in the ancestor of vascular plants.

Phylogenetic analysis of KDs enables differentiation of *LRR-RLK* subfamilies, but it does not provide information about the evolutionary relationships between the different subfamilies. Deeper nodes that represented phylogenetic relationships between different *LRR-RLK* subfamilies were not well-supported and varied between trees constructed by different methods, likely because the kinase domain is relatively short and conserved, and has relatively few informative characters. Therefore, the inter-subfamily relationships shown in Fig. 1 should be interpreted cautiously.

### Genomic structure of *LRR-RLK* genes

**Fig. 2:**
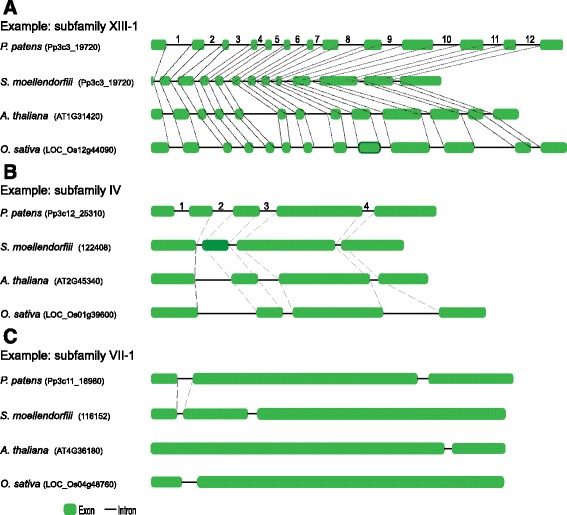
Three patterns of the evolution of *LRR-RLK* genes along major plant lineages. *Dashed lines* indicate conserved intron positions

**Fig. 3:**
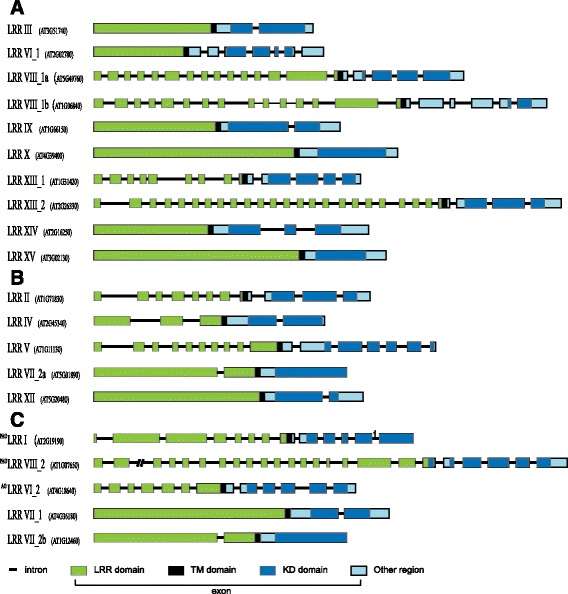
Intron/exon structure of representative genes of each subfamily. The intron/exon structures of representative genes of each subfamily were determined by comparison of the CDS with their corresponding genomic DNA sequences and were displayed using GSDS [43]. The IDs of representative genes of each subfamily are included in brackets. “AO” in the top left corner of a subfamily name indicates that members are only present in *A. thaliana* or *O. sativa*. “PAO” in the top left corner of a subfamily name indicates this subfamily members are only present in *P. patens*, *A. thaliana* or *O. sativa*, but not present in *S. moellendorffii*. **a** Subfamilies with intron/exon structures conserved in *P. patens*, *S. moellendorffii*, *A. thaliana*, and *O. sativa.* **b** Subfamilies with intron/exon structures conserved in *S. moellendorffii*, *A. thaliana*, and *O. sativa.* **c** Subfamilies with intron/exon structures were conserved in *A. thaliana* and *O. sativa*

We analyzed the intron/exon structures of *LRR-RLK* genes to try to answer two questions. (1) How did the intron/exon structures of each subfamily evolved along the major plant lineages? (2) Are gene structures conserved within subfamilies? To answer the first question, a comparison of *LRR-RLK* gene structures in *A. thaliana* and *O. sativa* with those of the same subfamilies in *P. patens* and *S. moellendorffii* was performed. According to the evolution of gene structures along the major plant lineages, *LRR-RLK* subfamilies were classified into three categories. In subfamilies of category A (Fig. 2a), genes from all four species shared the same gene structures (Fig. 2a and Additional file 2: Figure S1), suggesting that these common gene structures were established early in land plant evolution. For example, in subfamily XIII-1, 7 genes from *P. patens*, 1 gene from *S. moellendorffi*, 3 genes from *A. thaliana*, and 3 genes from *O. sativa* shared the same gene structure with 12 introns (Fig. 2a), which suggested that this common structure was established early in land plants and conserved during the evolution of different plant lineages. Another example was identified in subfamily IX, which consists of 13 genes: 2 genes from *P. patens*, 4 genes from *S. moellendorffi*, 4 genes from *A. thaliana*, and 3 genes from *O. sativa*. All genes in subfamily IX, except for one gene from *P. Patens*, showed the same simple gene structure with only one intron (Additional file 2: Figure S1). Although one subfamily IX member from *P. patens* (Pp3c15_17310) has two introns, one of its introns is identical to that of the other members of this subfamily. Furthermore, another gene from *P. patens* has only the same one intron as other members. These findings suggest that the one intron structure of subfamily IX was established early and conserved across different plant lineages; and the extra intron in one *P. Patens* gene may be specific to *P. patens*. Similarly, the same gene structures are shared by four species in members of *LRR* subfamilies III, VI-1, VIII-1, IX, X, XIII-1, XIII-2, XIV, and XV (Fig. 3a and Additional file 2: Figure S1). We used the structure of one *A. thaliana LRR-RLK* gene to represent the common gene structures shared by genes from all four species (Fig. 3a).

In subfamilies of category B (Fig. 2B), the same gene structure organization of the same subfamily only occurs in genes from vascular plants (*S. moellendorffii*, *A. thaliana*, and *O. sativa*) (Additional file 2: Figure S1)*.* The gene structure evolution of subfamilies II, IV, V, VII-2a, and XII belong to category B (Fig. 3b and Additional file 2: Figure S1). A comparison of the structures of *P. patens LRR-RLK* genes from these subfamilies with those of vascular plants revealed that *P. patens* genes have more introns in comparison with those of vascular plants. For example, all *LRR-RLK* genes of subfamily IV from vascular plants have three introns, whereas genes from *P. patens* contain four introns (Fig. 2b and Additional file 2: Figure S1), indicating that the ancestors of subfamily IV may have had four introns, one of which may have been lost during the evolution of vascular plants. For this kind of subfamily, most introns (which consist of the “basic gene structure”) were conserved during the evolution of different plant lineages and only a few ancestor introns were lost during the evolution of vascular plants. The conserved “basic gene structure” of each subfamily was shown with the structure of one *A. thaliana* gene (Fig. 3b).

In subfamilies of category C, the same gene structure organization is only shared by homologs from *A. thaliana* and *O. Sativa* or not shared by homologs from any of the four species (Fig. 2c). Subfamilies I, VI-2, VII-1, VII-2b, and VIII-2 belong to category C (Fig. 3c and Additional file 2: Figure S1). For subfamily VI-2, no homologs were found in *P. patens* and *S. moellendorffii*; indeed, they cannot share an intron/exon structure. Genes from subfamily I and VIII-2 are not present in *S. moellendorffii*, and genes from *P. patens* only shared some introns with genes from *A. thaliana* and *O. sativa.* For subfamily VII-1, although members can be found in all four species, members from *P. patens* and *S. moellendorffii* did not share introns with those from *A. thaliana* and *O. sativa* (Fig. 2c). Subfamily VII-2 can be divided into two subgroups (VII-2a and VII-2b), the evolutionary pattern of VII-2a belong to category B and that of VII-2b belong to category C.

The analysis described above revealed when the introns/structures of each subfamily originated, as well as how the gene structure of each subfamily evolved along different major plant lineages. To explore the conservation of gene structures in members within each subfamily, we calculated the proportions of introns shown in Fig. 3 and the proportions of genes with the structures shown in Fig. 3 in corresponding subfamilies. Among the 116 introns shown in Fig. 3a and b, 103 introns were present in more than 90% of the genes in a particular subfamily (Table 2). In addition, except four subfamilies, the proportions of genes from other subfamilies with structures shown in Fig. 3a and b were greater than 70%. This result suggested that most introns were conserved within subfamilies and most members of the same subfamily shared the common gene structure. In contrast, the proportions of some introns shown in Fig. 3c were relatively high and that of others are low, and the proportions of genes with structures shown in Fig. 3c were also lower, suggesting that the gene structures were less conserved in subfamilies of category C.

**Table 2:** Percentages of introns in Fig. 3 and percentages of genes with the same structures as genes in Fig. 3

Subfamily	Intron number	Percentages of presence of introns in Fig. 3	Percentage of gene
A			
III	1	P_i_ = 96.6%	P_g_ = 64.4%
VI-1	6	P_i1-4_ = 100%; P_i56_ = 72.7%	P_g_ = 54.5%
VIII-1a	18	P_i5-14,18_ = 100%; P_i1-4,12_ = 92.3%; P_i15,16_ = 76.9%	P_g_ = 69.2%
VIII-1b	19	P_i2-4,7,8,10-15,17_ = 100%; P_i1,6,9,18_ = 90.9%; P_i16,19_ = 81.8%	P_g_ = 54.5%
IX	1	P_i_ = 100%	P_g_ = 92.3%
X	0	P_i0_ = 75.9%	P_g_ = 75.9%
XIII-1	12	P_i2,4,6,7_ = 100%; P_i3,5_ = 94.4%; P_i1,8-10_ = 88.9%; P_i11,12_ = 83.3%	P_g_ = 72.2%
XIII-2	26	P_i2,4-26_ = 100%; P_i1,3_ = 90.9%	P_g_ = 72.7%
XIV	3	P_i1,2_ = 100%; P_i3_ = 90.9%	P_g_ = 81.8%
XV	0	P_i0_ = 83.3%	P_g_ = 83.3%
B			
II	10	P_i2-6,8_ = 100%; P_i1,7,10_ = 97.1%; P_i9_ = 94.3%	P_g_ = 77.1%
IV	3	P_i1-3_ = 100%	P_g_ = 77.8%
V	15	P_i1-3, 6–9, 13_ = 100%;P_i4–5,12,15_ = 96%;P_i14_ = 92%; P_i10,11_ = 80%;	P_g_ = 60%
VII-2a	1	P_i_ = 100%	P_g_ = 70%
XII	1	P_i_ = 96.6%	P_g_ = 83%
C			
I^PAO^	12	P_i12_ = 100%; P_i3–5, 9–11_ = 98.4%; P_i7_ = 92.1%; P_i1,2_ = 90.4%;	P_g_ = 42.9%
VIII-2^PAO^	23	P_i1-3,19–22_ = 100%; P_i5,6,8,12,18_ = 97.7%;P_i23_ = 95.5%; P_i4, 7, 9–11, 13–17_ = 65.9% ~ 86.4%	P_g_ = 56.8%
VI-2^AO^	11	P_i1–11_ = 100%	P_g_ = 50%
VII-1	1	P_i_ = 22.2%	P_g_ = 22.2%
VII-2b	1	P_i_ = 37.5%	P_g_ = 37.5%

AO indicates that members are only present in *A. thaliana* or *O. sativa*. PAO indicates that members are only present in *P. patens*, *A. thaliana* or *O. sativa*, but not present in *S. moellendorffii*

For most subfamilies from category A and B, the common gene structures or basic gene structures were established in early land plants. These gene structures are conserved within subfamilies and across different plant lineages, but divergent among subfamilies (Fig. 3). In contrast, gene structures from category C subfamilies are neither conserved across different lineages nor within subfamilies. The common gene structures of subfamilies III, VI-1, VIII-1, IX, X, XIII-1, XIII-2, XIV, and XV contain 1, 6, 19/18, 1, 0, 12, 26, 3, and 0 introns, respectively (Fig. 3a and Additional file 3: Table S2). The basic gene structures of subfamilies II, IV, V, VII-2a, and XII contain 10, 3, 15, 1 and 1 introns (Fig. 3b and Additional file 3: Table S2), respectively.

### Conserved motifs

To further investigate the protein evolution of *LRR-RLK* genes, the conserved motifs of extracellular domains containing LRR and KD domains were identified with Multiple Expectation Maximization for Motif Elicitation (MEME) program v.4.10.2 [51]. LRR repeats are generally 20–29 residues long and can be classified into seven distinct subfamilies based on their conserved sequences [57]. The typical length of plant-specific LRR subfamily is 24 residues and their consensus sequence is LxxLxxLxLxxNxLxGxIPxxLxx [57]. We identified 16 LRR motifs in the extracellular domain. The basic LRR motif was L/cxxLxLxxNxL/fsGxI/lPxxL/Ixx (Table 3), which matches well with the plant LRR consensus sequence. The most conserved amino acid residues were Asn at position 9, Gly at position 16, and Pro at position 19, but Leu residues at positions 4, 7 and 9 were also well conserved. Among these motifs, L1 and L2 were shared by all subfamilies and almost all members of each subfamily (Additional file 3: Table S2). Motifs L3 and L4 appeared in all subfamilies except for subfamily I and VI-2. Motif L6 was present in all subfamilies other than I, II, IV, VI-2, XIII-1. Motifs L7 mainly appeared in subfamilies VI-1, VII-1, VII-2, VIII-1, VIII-2, X, IX, XII, XIII-2, XIV and XV. Motif L8 mainly appeared in subfamilies VII-1, VII-2, VIII-1, X, XI, XII, XIII-2 and XV. Motifs L9, L10, L11, L12, L13 were shared by all members of subfamilies VII-1, VII-2, X, XI, XII, XIII-2 and XV. Motifs L15, L17, L18 and L19 were shared by almost all members of subfamilies VII-1, X, XI, XII, and XIII-2. In total, the result showed that most of the closely related members in the phylogenetic tree had similar motifs and similar arrangements of the different LRR motifs, whereas members of different subfamilies usually contained different LRR motif compositions. The motif arrangements of some subfamilies with mostly identical LRR motifs were different. For example, subfamilies II and IV both contained LRR motifs L1, L2, L3, L4 and L15; the arrangement of LRR motifs in subfamily II is L15, L3, L1/L2 and L4, while the arrangement of that in subfamily IV is L15, L3, L2 and L1/L4 (Additional file 3: Table S2). In addition to LRR motifs, four non-LRR motifs (L5, L14, L16 and L20) were also identified in the extracellular regions of LRR-RLK proteins (Additional file 4: Table S3). L5 and L16 occurred in most subfamilies, L14 occurred in some subfamilies, whereas L20 only occurred in subfamily I.

**Table 3:** Major motifs in the predicted LRR domains of LRR-RLKs

Motif	20	21	22	23	24	1	2	3	4	5	6	7	8	9	10	11	12	13	14	15	16	17	18	19	20	21	22	23	24	1	2
L1	x	x	L	x	x	**L**	x	x	**L**	x	x	**L**	D	**L**	**S**	x	**N**	x	**L**/f	t/s	**G**	x	**I**	**P**							
L2	x	x	L	g	x	L	x	x	**L**	x	x	**L**	d	**L**	S	x	**N**	x	**L**/f	S/t	**G**	x	**I**	**P**							
L3	x	x	L/i	L	x	L	x	x	**L**	x	x	**L**	x	**L**	x	x	**N**	x	L/f	t/s	**G**	x	**I**/l	**P**							
L4	x	x	L	g	x	**L**	x	x	**L**	x	x	L	x	L	S	x	**N**	x	x	S	**G**	x	**I**	**P**							
L6								x	**L**	x	x	L	x	L	S	x	**N**	x	**L**/f	t/s	**G**	x	I	**P**	x	x	l	x	x	x	x
L7	x	x	L/i	g	x	C	x	x	**L**	x	x	**L**	x	**L**	x	x	**N**	x	L/f	x	**G**	x	I/l	**P**							
L8	x	x	L/i	G	x	**L**	x	x	**L**	x	x	**L**	x	**L**	x	x	**N**	x	L/f	s	**G**	x	I/l	**P**							
L9	p	x	L/i	G	n	L	t	x	**L**	x	x	**L**	x	L	s	x	**N**	x	L/f	x	**G**	x	I/l	**P**							
L10	x	x	L/i	x	x	C	x	x	**L**	x	x	L	x	L	x	x	**N**	x	**L**/f	x	**G**	x	I/l	**P**							
L11	x	x	l	x	x	L	x	x	**L**	x	x	**L**	d/n	**L**	S	x	**N**	x	L/f	x	**G**	x	I/l	**P**							
L12								x	**L**	x	x	L	x	L	s	x	**N**	x	F/L	t	**G**	x	l/i	**P**	x	x		x	x	l	x
L13	x	x	l/L	x	x	L	x	x	**L**	x	x	**L**	x	L	x	x	**N**	x	L/f	t/s	**G**	x	I/l	**P**							
L15	x	x	L/	x	x	C	x	x	**L**	x	x	L	x	**L**	x	x	**N**	x	L/f	s	**G**	x	L/i	**P**							
L17	x	x	l	G	x	L	x	x	**L**	x	x	**L**	x	L	x	x	**N**	x	**L**	x	**G**	x	I	**P**							
L18	x	x	l	x	x	L	x	x	**L**	x	x	L	x	L	s	x	**N**	x	**E**	x	**G**	x	I	**P**							
L19	x	x	x	x	x	x	x	x	l	x	x	**L**	x	**L**	S	x	**N**	x	L/f	t/	**G**	x	I/l	**P**							

If the bits value of the amino acid at this position is smaller than 0.5, it is represented with x; 1 > bits ≥ 0.5, with a lowercase letter; 2 > bits ≥ 1, with a capital letter; 3 > bits ≥ 2, with a bold capital letter; bits ≥ 3, with an underlined bold capital letter

The KD of eukaryotic protein kinases contains 250 − 300 amino acid residues and is divided into 12 smaller subdomains (I–XII) [4, 5]. These subdomains usually contain conserved residues [4, 5]. The *LRR-RLK* KD contains approximately 250–280 amino acid residues. MEME analysis identified the following 20 motifs in the *LRR-RLK* KD from the N-terminus to the C-terminus: Q-M3, Q-M4, Q-M1, Q-M2, Q-M5, Z-M2, Z-M1, Z-M5, Z-M3, Z-M4, H-M1, H-M3, H-M10, H-M9, H-M4, H-M5, H-M6, H-M7, H-M8, and H-M2 (Table 4). Based on conserved amino acids, motifs Q-M3, Q-M4, Q-M1, Z-M1, Z-M3, H-M1, and H-M2 correspond to subdomains I, II, III, VIb &VII, VIII, IX, and XI, respectively. These motifs, except for motif VIII (Z-M3) and four other motifs (Q-M2, Z-M2, Z-M4 and H-M4), are shared by all subfamilies and almost all members of each subfamily. Motifs Q-M2 and Z-M2 are contained within subdomains V and VIa according to the amino acid alignment. Motifs Z-M3, Z-M5, and H-M3 were identified in different *LRR-RLK* subfamilies. For example, motif Z-M3 was absent from all *LRR-RLK* genes of subfamilies VI-1 and VI-2, as well as most of those of subfamily XIV. Motif H-M3 was not observed in any *LRR-RLK* genes of subfamilies VI-1and XIV and in most genes of subfamilies IV and VII-2. We also identified subfamily-specific motifs. For example, motif H-M5 appeared only in subfamily I, motif Q-M5 appeared only in subfamily XII, and motifs H-M9 and H-M7 appeared only in subfamily V.

**Table 4:** Major motifs in the predicted kinase domains of LRR-RLKs

Subdomains	Motifs	Sequences
I	Q_M3	**G**x**G**gf**G**x**V** **Y**K/rA/GxLxd
II	Q_M4	GxxV**A** **V**/i**K**rLxxxxxx
III & IV	Q_M1	x**E**vex**L**/igxv/ir**H**r**NL**/i**V**x**L**x**G**YC
V	Q_M2	**L**V**Y**E/dY/fMpN**G**S**L**xxx**L**
	Q_M5	S/ti**D**xx**G**ND/E**F** **K** **A**
VIa	Z_M2	**W**xx**R**lx**I**AlG/da/v**A**r**G**/a**L**x**Y** **LH**xx
VIb & VII	Z-M1	PxIv/i**H**R**D**i/v/l**K**sS**N** **I**/v**LL** **D**xxfeA/pkV/i/la/s**D** **F** **G** **L**A/sk/r
	Z_M5	xxxxx**T**/s**H**V
VIII	Z_M3	stxva**G**Tx**GY**i/lA**PE** **Y**
	Z_M4	xT/sxKs**DV** **Y**/f
IX	H_M1	Ks**DV** **Y**/f**SF**/y**G** **V**/iV/l**L** **LE**Ll/v/i**TG**k/rxPx
	H_M3	xxxxxL/ivx**W**V/a
	H_M10	eYx**E**d/e**D**V**V**i/v**L**c**D**h**V** **R**/k
	H_M9	**P** **QLHDI**
	H_M4	xxxxxv/iv**D**pxL
	H_M5	gd**Y**Dxx**S**V**W** **K**/ra/v
	H_M6	xxxEeE**M**v/lxv**L**
	H_M7	x**YP**A**KSLS** **R** **FA**
	H_M8	eY/Fxxx**E**V/axrm/vI
XI	H_M2	xl/i/v**A**/glx**C**txxx**P**xx**RP**x**M**xe**V**V

If the bits value of the amino acid at this position is smaller than 0.5, it is represented with x; 1 > bits ≥ 0.5, with a lowercase letter; 2 > bits ≥ 1, with a capital letter; 3 > bits ≥ 2, with a bold capital letter; bits ≥ 3, with an underlined bold capital letter

### Selection test

UP clusters (related only by duplication) and SO clusters (related only by speciation) were identified as reported in Fischer et al. [23] using a tree reconciliation approach [53]. All SO clusters identified in the present study had three or less sequences. This finding was expected because the number of sequences that a SO cluster could contain was at most four (the number of species used in this study). As a minimum of four sequences was required in the site-model analysis, all SO clusters were ignored in subsequent selection analyses. Only UP clusters containing five or more sequences were considered in the analysis. After cleaning, the final data set comprised 20 UP clusters (Table 5). To evaluate the selective pressures acting on these UP clusters, we conducted likelihood ratio tests using three pairs of models (Table 5). The LRTs for model M3 *versus* model M0 were significant in all cases, indicating that ω was variable among sites along the *LRR-RLK* sequences in all UP clusters (Table 5). Models M2 and M8 assume positive selection, whereas models M1 and M7 are nearly neutral. Both LRTs for model M2 *versus* model M1 and model M8 *versus* model M7 suggested that positive selection occurred at sites within 6 UP clusters (Table 5): 1, 2, 6, 11, 15 and 16. In addition, tests on models M8 and M7 detected sites of positive selection within 3 UP clusters: 5, 9 and 17. Nine UP clusters evolved under positive selection, accounting for 45% UP clusters. As shown in Table 5, all 9 UP clusters with codons under positive selection come from four subfamilies: I, IIII, VIII-2 and XII. For UP clusters other than these nine UP clusters, models M2 and M8 were not significantly better than models M1 and M7, and no site was found to be under positive selection by Bayes empirical Bayes inference using a probability criterion of 90. Therefore, the nearly neutral model most closely simulated the observed data for these subfamilies. In model M1, the ω value ranged from 0.02 to 0.71 for codons of these *LRR-RLK* UP clusters, suggesting purifying selection of codons.

**Table 5:** Likelihood ratio test of positive selection in LRR-RLK subfamily proteins

UP cluster	Subfamily	2 L/M3 vs. MO	2 L/M2a vs. M1a	2 L/M8 vs. M7	M8 estimates^a^	Positively selected sites (posterior > 0.90)^b^
1	I	5379.61^***^	80.22^***^	47.55^***^	p1 = 0.040, ω = 1.43	56, 138, 242, 359, 365, 375, 387, 638
2	I	1462.2^***^	21.08^***^	28.26^***^	p1 = 0.032, ω = 3.12	110, 349,417
3	II	198.35^***^	0.05	1.86	p1 = 0.003, ω = 12.76	none
4	III	280.93^***^	0	2.29	p1 = 0.012, ω = 998.45	none
5	III	212.91^***^	0	6.52*	p1 = 0.014, ω = 9.76	390 (>80)
6	III	451.92^***^	18.39^***^	28.59^***^	p1 = 0.165, ω = 2.38	92, 126, 179, 202, 335, 351
7	V	446.10^***^	0	0.44	p1 = 0.011, ω = 2.94	none
8	VI-1	246.12^***^	0	3.13	p1 = 0.001, ω = 5.75	none
9	VIII-2	1215.03^***^	0	17.38^***^	p1 = 0.013, ω = 45.27	186, 274,
10	VIII-2	660.99^***^	0	5.69	p1 = 0.011, ω = 998.64	none
11	VIII-2	2093.24^***^	45.59^***^	72.78^***^	p1 = 0.065, ω = 1.67	35, 39, 45, 164, 1060, 1102, 1107
12	X	902.09^***^	0	0.56	p1 = 0.001, ω = 98.59	none
13	X	2235.18^***^	0	3.46	p1 = 0.025, ω = 1.02	none
14	XII	294.04^***^	0	5.05	p1 = 0.021, ω = 203.11	none
15	XII	345.23^***^	10.55^**^	25.08^***^	p1 = 0.046, ω =3.07	357, 408, 430
16	XII	2781.61^***^	23.30^***^	32.12^***^	p1 = 0.012, ω = 1.00	370, 468, 470
17	XII	4290.61^***^	3.95	6.42^*^	p1 = 0.002, ω = 138.05	409
18	XIII-1	437.0^***^	0	4.92	p1 = 0.055, ω = 1.51	none
19	XIII-1	226.25^***^	0	0.069	p1 = 0.001, ω = 4.60	none
20	XIV	388.89^***^	0	1.35	p1 = 0.006, ω = 111.67	none

*:significant at 0.05% level; **:significant at 0.01% level; ***:significant at 0.001% level

^a^ω is dN:dS estimated under M8 model; p1 is the inferred proportion of positively selected sites

^b^Sites potentially under positive selection identified under model M8 are listed according to conserved sequence numbering. Positively selected sites in LRR motifs are underlined

## Discussion

### Expansion of the *LRR-RLK* gene family in Viridiplantae

Our study identified 119 *LRR-RLK* genes in the *Physcomitrella patens* moss genome, 67 *LRR-RLK* genes in the *Selaginella moellendorffii* lycophyte genome, and no *LRR-RLK* genes in five green algae genomes (*Chlamydomonas reinhardtii*, *Micromonas pusilla* CCMP1545 and *Micromonas* sp.RCC299, *Ostreococcus lucimarinus*, and *Volvox carteri*) (Additional file 1: Table S1). *LRR-RLK* genes contain a LRR and a KD. It has been proposed that domain-shuffling events may lead to the founding of *RLK* subfamilies [1]. LRRs and KDs are present in all genomes, including those of green algae and other plants [45]. *LRR-RLK* genes were not detected in green algae, but their presence in land plants suggests that the structural combination of LRRs and KDs to form new genes may have occurred after the divergence of land plants from the green algae. Previous studies have identified *LRR-RLK* genes from eight angiosperms with copy numbers ranging from 213 in *A. thaliana* to 467 in *Glycine max* (Table 1). A recent study reported there are 7,554 *LRR-RLK* genes in 31 fully sequenced flowering plant genomes, with an average of 243 *LRR-RLK* genes in each angiosperm genome [23]. Hence, although the *P. patens* and *S. moellendorffii* genomes contain *LRR-RLK* genes, while the green algae genomes do not, there are substantially fewer *LRR-RLK* genes in moss and lycophytes than in higher (flowering) plants. Differences in the copy numbers of *LRR-RLK* genes in moss, lycophytes and angiosperms may be due to the different expansion rates of *LRR-RLK* genes in different genomes, but may also be due to the difference in genome sizes. To distinguish these factors, we compared the proportions of *LRR-RLK* genes among all protein-coding genes in different genomes. The percentage of *LRR-RLK* genes in moss is 0.36%, while that in *S. moellendorffii* is 0.30% (Table 1, no significant difference). However, the percentages of *LRR-RLK* genes in these two species are much lower than that in angiosperms, which ranges from 0.67 to 1.39%. These results indicate that *LRR-RLK* genes in Viridiplantae have undergone a large degree of expansion in the lineages leading to the flowering plants. Earlier studies suggest that the *RLK* gene superfamily underwent extensive expansion in land plant lineages, primarily due to the expansion of a few families [46, 58]. In good agreement with previous studies, the expansion of the *LRR-RLK* family, which is a major group of plant *RLK*s, contributed to the expansion of *RLK* genes through both adaptive and non-adaptive evolution [46, 58]. *LRR-RLK* genes have important roles in development and defense responses, and continuous selection pressure imposed by the developmental complexity of flowering plants and changing environmental stimuli might be responsible for the expansion of this gene family. Alternatively, expansion of *LRR-RLK* genes may reflect random genomic drift, as functional redundancy is common among *LRR-RLK* genes [59, 60].

### Origin, gene structure, and protein sequence evolution of each *LRR-RLK* subfamily

According to the tree topologies and clade support values, LRR-RLK genes were classified into 19 subfamilies. The subfamily definitions were supported not only by the phylogenetic analysis, but also by the unique gene structures (unique basic gene structures, Fig. 3), and the protein motif compositions of each subfamily (Additional file 3: Table S2 and Additional file 4: Table S3). Gene structures and protein motifs will be discussed in subsequent paragraphs. The phylogenetic trees (Fig. 1 and Additional file 2: Figure S1) show that all subfamilies included sequences from *A. thaliana* and *O. sativa*, and all subfamilies except one (VI-2) also contained *LRR-RLK* gene sequences from *P. patens*. Among the 18 subfamilies that included *P. patens LRR-RLK* sequences, two subfamilies (I and VIII-2) lacked *S. moellendorffii* sequences. Using the most parsimony assumption, the ancestors of subfamilies I and VIII-2 likely evolved from the common ancestor of land plants before the divergence of specific lineages, which were subsequently lost in the lycophyte. Therefore, most *LRR-RLK* subfamilies (18 of 19, or 95%) were established early in land plant evolution before the divergence of moss and other land plant lineages. In addition, in subfamilies II, III, VII-2, VIII-1, X, and XI, several clades include sequences of *P. patens*, *S. moellendorffii*, *A.thaliana* and *O.sativa*, and this is the opposite situation for other subfamilies. The result could be interpreted as contrasted ancestral copy number between subfamilies. Namely, for these subfamilies, there were probably several *LRR-RLK* genes before the split between *P. patens*, *S. moellendorffii* and angiosperms. The early origin of most subfamilies indicates that genes in most subfamilies may have central roles in the regulation of common developmental and defense pathways of different land plant lineages. Some subfamily members with specific developmental roles, such as the control of pollen tube development (PRK in subfamily III) [61] and vascular development (PSY in subfamily XI) [62], were established in early land plants. Many of the gene families that control the development of flowering or vascular plants were present in early land plants [63]. Further studies are needed to investigate the specific functions of each member of these gene families. There are no subfamily VI-2 members in *P. patens* or *S. moellendorffii*; this subfamily is only found in *A. thaliana* (e.g., *MRH1*) and *O. sativa*, indicating that it evolved recently in higher plants. *MRH1* is required specifically for root hair elongation growth [64]. The absence of *MRH1* homologs in moss may reflect the fact that mosses possess rhizoids. The absence of *MRH1* homologs in the lycophyte *S. moellendorffii* suggests that root hair growth may be regulated differently in lycophytes and flowering plants.

Eukaryotic genes usually contain introns. The ancestor genes that emerged to establish each subfamily evolved protein-coding exons and introns between the exons. To elucidate the evolution of the intron/exon structure of each subfamily, we analyzed the structures of *LRR-RLK* genes. For nearly half of the subfamilies (LRR III, VIII-1, IX, X, XIII-1, XIII-2, XIV and XV), identical intron/exon gene structures in the same subfamily were found in *P. patens, S. moellendorffii*, *A. thaliana* and *O. sativa*; these gene structures were shared by the majority members of each subfamily (Figs. 2a and 3a, Table 2 and Additional file 2: Figure S1). These results suggest that the intron/exon structures of these subfamilies (category A) were established before the divergence of mosses and vascular plants, and they were evolutionarily conserved following plant evolution from moss to flowering plants. Meanwhile, we found that the gene structures of other subfamilies (category B: II, IV, V, VII-2a and XII; Figs. 2b and 3b, Table 2 and Additional file 2: Figure S1) were relatively less conserved across different plant lineages. The *P. patens* gene sequences of these subfamilies usually have additional introns beyond those characteristic of the basic gene structure of their particular subfamily (Fig. 2b and Additional file 2: Figure S1). The additional introns in these species may represent ancestral introns that were lost during vascular/flowering plant evolution or introns that were gained after the divergence of these lineages from other plants. Except for the extra introns in *P. patens* gene sequences, the high percentages of presence of introns (Table 2) suggested that most introns comprising the basic gene structure in these subfamilies were conserved. Therefore, it is clear that, for most subfamilies from category A and B, most *LRR-RLK* introns are conserved within each subfamily and across different plant lineages. Intron sequences are subject to selection not only because they may contain ORFs or form part of coding sequecnes due to alternative splicing, but also because they can play a regutory role in transcription or translation, or in maintaining pre-mRNA secondary structure [24, 65]. These diverse roles may explain why intron positions are highly conserved in many other genes [66, 67] and gene families [68, 69]. With regard to *LRR-RLK* genes, a previous study demonstrated that multiple introns of *LRR-RLK* gene *ERECTA* are essential for its expression in *A. thaliana* [25]. The genome structural conservation of *LRR-RLK* subfamilies suggests that gene diversification within subfamilies could be under strong selection pressure and indicative of their functional conservation.

The gene structures or basic gene structures are conserved within most subfamilies and across land plants (Table 2 and Additional file 2: Figure S1), but they diverge among different subfamilies (Fig. 3). Each subfamily has a unique gene structure or unique basic gene structure (Fig. 3). The structures of each subfamily provide additional evidence to support the subfamily classifications and, more importantly, indicate the potential functional divergence. Although most introns are conserved during plant evolution and gene duplications, some intron gains and losses occur (Table 2 and Additional file 2: Figure S1). In addition, the genomic structures of the *LRR-RLK* genes of some subfamilies are not conserved in moss, lycophyte and angiosperm species (Fig. 2c), suggesting more prevalent intron gain and loss events. Differences between subfamilies with regard to numbers of intron gains and losses may be indicative of their degree of functional divergence.

Protein function is linked to the protein sequence. The analyses of conservation and variation in protein sequences supported the functional divergence of different LRR-RLK subfamilies. LRR-RLK proteins contain three functional domains: the LRR domain, a transmembrane domain, and an intracellular kinase domain. The LRR is a widespread structural motif of 20–29 amino acids with conserved leucine. The typical length of plant LRRs is 24 amino acids. LRR-RLKs contain variable numbers and arrangements of LRRs (1–30) [45]. In the present study, we identified 16 LRR motifs with a length of 24 residues in the LRR domain. The basic motif was L/cxxLxLxxNxL/fsGxI/lPxxL/Ixx (Table 3), which matched well with the plant LRR consensus sequence (LxxLxxLxLxxNxLxGxIPxxLxx) [57]. Previous studies reported that members of the same LRR-RLK subfamily tend to have similar LRR structural arrangements, whereas members of different subfamilies exhibit different LRR numbers and arrangements [1, 18]. This pattern remained after *P. patens* and *S. moellendorffii LRR-RLK* sequences were included in the analysis (Additional file 3: Table S2)*.* LRRs directly influence ligand binding. The diversity of LRRs allows RLKs to respond to a variety of extracellular signals, including small protein ligands, such as plant-derived ClV3 or flagellin, which is derived from microbes [70]. Hence, the divergence of LRRs among different subfamilies appears to reflect their divergence with respect to ligand perception.

When the LRR domain binds a ligand, the KD is activated to trigger the subsequent activation of downstream substrates [2]. The conserved KD of eukaryotic protein kinases is divided into 12 smaller subdomains (I–XII); these subdomains generally contain characteristic patterns of conserved residues (except for subdomains IV, V, and X) [4, 5]. Crystal structures and mutation analyses demonstrated that these conserved residues play essential roles in enzyme function [4, 5, 71]. Although most KDs are relatively conserved, functional divergence of the KD region has been reported in some LRR-RLKs [72]. Our MEME motif analysis identified 11 motifs (Table 4) that are shared by all subfamilies and essentially all members of each subfamily. Seven of these motifs correspond to the seven subdomains with conserved amino acids (I, II, III, VII, VIII, IX, and XI). The common motifs of LRR-RLK proteins in different subfamilies may suggest their functional similarities. However, the MEME analysis also showed that some motifs are limited to some subfamilies, implying that functional diversification of KDs occurred among subfamilies. For example, subdomain VIII (motif Z-M3) contains a highly conserved triplet APE that is required for kinase activity [4, 5]. The absence of this motif from subfamilies VI-1, VI-2, and XIV may suggest large functional changes in these subfamilies. Another example is the Z-M5 motif position between subdomains VII (with conserved triplet DFG) and VIII (with conserved triplet APE). This region usually contains Ser/Thr residues, and phosphorylation of these sites is essential for catalytic activation of some LRR-RLKs [3, 5, 73]. The absence or presence of this motif in some subfamilies may influence their regulatory effect on enzymatic activity. Furthermore, we identified subfamily-specific motifs (Table 4). Motifs H-M5, H-M10, and Q-M5 appear only in subfamilies I, VII-2, and XII-2, respectively, whereas motifs H-M9 and H-M7 are present only in subfamily V. These subfamily-specific motifs may contribute to the functional divergence of different subfamilies.

### Positive selection contributed to the evolution of certain subfamilies

Substitutions can change the functions of duplicated genes in gene families, and may be due to a relaxation of purifying selection or the action of positive selection [34]. To investigate the relative contributions of relaxation of purifying selection *versus* positive selection in the evolution of *LRR-RLK* subfamilies, we performed selection pressure tests. Recent studies demonstrated that orthologs and paralogs in gene families evolve under different levels of positive selection pressure [23, 52]. Therefore, in this study, we first identified UP clusters and SO clusters as reported in Fischer et al. [23] using a tree reconciliation approach [53], after which we estimated the ω value of the genes of each cluster. All SO clusters identified in the present study had three or less sequences, so they were ignored in subsequent selection analyses. However, among the 20 UP clusters identified in this study, 9 (45%) contained codons under positive selection (Table 5), which is consistent with a previous report that positive selection is prevalent at lineage-specific expanded genes (paralogs) of *LRR-RLK* genes in angiosperm, 50% of which contained codons under positive selection pressure [23]. Hence, our results suggest that the findings of Fischer et al. [23] remain true when *LRR-RLK* genes from more basal plants are considered . Moreover, our results are largely consistent with the findings of Fischer et al. [23] at a subfamily level. Fischer et al. [23] found that all UP clusters with codons under positive selection pressure came from subgroups I, VIII-2, and XII (a and b) [23]. In our study, we detected 4 subfamilies (I, III, VIII-2 and XII) under positive selection pressure, of which three subfamilies (I, VIII-2 and XII) are the same as thosed identified by Fischer et al. [23]. Therefore, at the subfamily level, positive selection may have driven the evolution of only a few subfamilies, Sun and Wang [17] also suggested that positive selection only contributed to the evolution of a few *LRR-RLK* subfamilies defined in *O. sativa*.

The positively selected sites were located primarily in the *LRR* region of *LRR-RLK* genes (Table 5). This result is consistent with the study of Fischer et al. [23] which found that most codons under selection fall in the LRR domain. The LRR domain occurs in diverse proteins, particularly in many proteins involved in defense responses. Positive selection shapes the LRR domains to generate new pathogen-recognition specificities [35]. In the present study, we found that, among the four subfamilies in which positively selected sites were identified, the functions of genes from subfamily VIIII-2 are not known, whereas the functions of several members from the other three subfamilies (I, III and XII) are well characterized. Subfamily III usually contains genes involved in development, while genes from subfamilies I and XII are usually involved in defense. Subfamily I members include the *IOS1* and *FRK* genes, which are involved in defense signaling [74], Subfamily XII members include *FLS2* and the *EF-Tu Receptor* (EFR) (from *A. thaliana)*, which are involved in innate immunity against pathogens [12], as well as *O. sativa* Xa 21, which is involved in resistance to bacterial pathogen *Xanthomonas oryzae* pv. oryzae [75]*.* Furthermore, *Xa 21* was found to have evolved under positive selection in rice [36, 37], and *FLS2* showed a signature of rapid fixation of an adaptive allele in *A. thaliana* [38]. The detection of positive selection in these two subfamilies is consistent with their roles in plant defense.

## Conclusions

The evolutionary relationships among *LRR-RLK* genes have been investigated in flowering plants. However, due to the lack of phylogenetic analysis of *LRR-RLK* genes from diverse plants, including algae, bryophytes, and different lineages of vascular plants, the classification of *LRR-RLK* genes in plants, and the origin, gene structure, and protein motif evolution, and the force driving the evolution of each *LRR-RLK* subfamily remain to be understood. Our studies identified 119 *LRR-RLK* genes in the *Physcomitrella patens* moss genome, 67 *LRR-RLK* genes in the *Selaginella moellendorffii* lycophyte genome, and no *LRR-RLK* genes in five green algae genomes. Phylogenetic analyses from these sequences and sequences from two flowering plant species revealed that plant *LRR-RLK*s belong to 19 subfamilies, most of which were established in the common ancestors of land plants. More importantly, we found that each subfamily was characterized by unique gene structures or unique basic gene structure and protein motif compositions. Four subfamilies were found to be under positive selection. Taken together, these results provide strong evidence that functional divergence occurred among *LRR-RLK* subfamilies and that positive selection had only an impact on the evolution of a few subfamilies of *LRR-RLK* genes.

## Additional files

Additional file 1: Table S1.
*LRR-RLK* genes identified in *Physcomitrella patens* and *Selaginella moellendorffii*. (XLS 4566 kb)
Additional file 2: Figure S1. Phylogeny of *LRR-RLK* genes. This phylogenetic tree based on kinase domain sequence was constructed by the maximum likelihood method based on kinase domain sequences. Subfamily names are shown on the right. The intron/exon structure of each LRR-RLK gene and intron gain and loss eventS were mapped onto the tree. (PDF 8177 kb)
Additional file 3: Table S3. Schematic illustrations of the types and distribution of LRR motifs in each LRR-RLK subfamily. (XLS 884 kb)
Additional file 4: Table S4. Non-LRR motifs identified in the extracellular regions of LRR-RLK proteins. (DOC 27 kb)
Additional file 5:
**Data S1.** Alignment of the kinase domains of putative LRR-RLK sequences. (FAS 577 kb)

## Abbreviations

ECD: An extracellular domain
KD: Kinase domain
LRR-RLK: Leucine-rich repeat receptor-like protein kinases
RLK: Receptor-like kinases
