# Supplementary figures and images for: Origin and diversification of leucine-rich repeat receptor-like protein kinase (LRR-RLK) genes in plants
Source: BMC Evol Biol. 2017 Feb 7;17:47. doi: 10.1186/s12862-017-0891-5 (PMC5296948; doi:10.1186/s12862-017-0891-5)

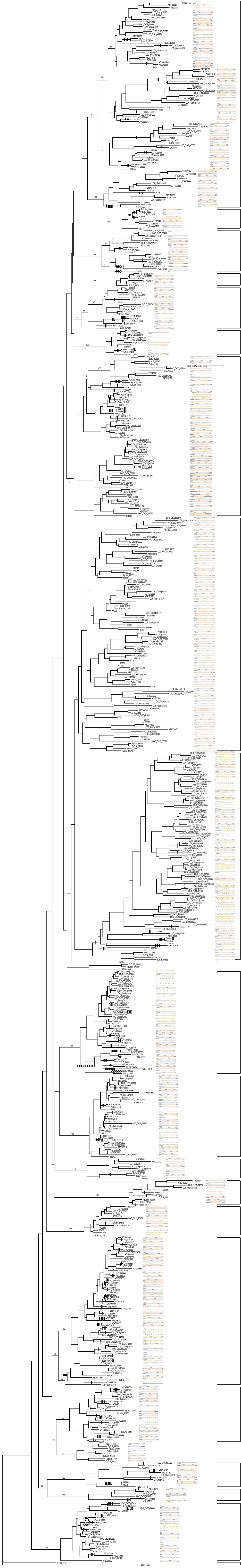

III

VII-1

VII-2

XV

XIII-1

XIII-2

X

XI

XII

VIII-2

II

IV

XIV

IX

I

VIII-1

VI-1

VI-2

V

outgroup

Supplement: Additional file 2: Figure S1. — Phylogeny of LRR-RLK genes. This phylogenetic tree based on kinase domain sequence was constructed by the maximum likelihood method based on kinase domain sequences. Subfamily names are shown on the right. The intron/exon structure of each LRR-RLK gene and intron gain and loss eventS were mapped onto the tree. (PDF 8177 kb) [file 12862_2017_891_MOESM2_ESM.pdf]
