# Supplementary material for: Origin and diversification of leucine-rich repeat receptor-like protein kinase (LRR-RLK) genes in plants
Source: BMC Evol Biol. 2017 Feb 7;17:47. doi: 10.1186/s12862-017-0891-5 (PMC5296948; doi:10.1186/s12862-017-0891-5)
Supplement: Additional file 4: Table S4. — Non-LRR motifs identified in the extracellular regions of LRR-RLK proteins. (DOC 27 kb) [file 12862_2017_891_MOESM4_ESM.doc]

| L5 | XxxxxxxxxCx**W**x**GV**/i**C**xxxxxxx |
| --- | --- |
| L14 | FxxxxxxsFx**GN**xx**LCG**xxxxxxc |
| L16 | XxxxxxxxD/ExxA**L**lxf/lKxxlxxxx |
| L20 | **C**YxlxV/lxxGxk**YLIR**At**F**x**YGNYD** |
